# Lean Neural Networks for Autonomous Radar Waveform Design

**DOI:** 10.3390/s22041317

**Published:** 2022-02-09

**Authors:** Anthony Baietto, Jayson Boubin, Patrick Farr, Trevor J. Bihl, Aaron M. Jones, Christopher Stewart

**Affiliations:** 1 Department of Computer Science and Engineering, The Ohio State University, Columbus, OH 43210, USA; boubin.2@osu.edu (J.B.); cstewart@cse.ohio-state.edu (C.S.); 2 Applied Research Solutions, Beavercreek, OH 45440, USA; patrick.farr.ctr@us.af.mil; 3 Sensors Directorate, Air Force Research Laboratory, Wright-Patterson Air Force Base, OH 45433, USA; trevor.bihl.2@us.af.mil (T.J.B.); aaron.jones.41@us.af.mil (A.M.J.)

**Keywords:** radar, neural network, optimization

## Abstract

In recent years, neural networks have exploded in popularity, revolutionizing the domains of computer vision, natural language processing, and autonomous systems. This is due to neural networks ability to approximate complex non-linear functions. Despite their effectiveness, they generally require large labeled data sets and considerable processing power for both training and prediction. Some of these bottlenecks have been mitigated by recent increased availability of high-quality data sets, improvements in neural network development software, and greater hardware support. Due to algorithmic bloat, neural network inference times and imprecision make them undesirable for some problems where fast classical algorithm solutions already exist, other classes of algorithms, such as convex optimization, with non-trivial execution times could be reduced using neural solutions. These algorithms could be replaced with light-weight neural networks, benefiting from their high degree of parallelization and high accuracy when properly trained. Previous work has explored how low size, weight, and power (low SWaP) neural networks and neuromorphic computing can be used to improve autonomous radar waveform design techniques that currently rely on convex optimization. Autonomous radar waveform design helps meet the need for interference mitigation caused by an ever-growing number of consumer and commercial technologies which pollute the radio frequency (RF) spectrum. Spectral notching, a radar waveform design technique, augments transmitted radar waveforms to avoid frequencies with excessive interference while maintaining the integrity of the waveform. In this paper, we extend that work, demonstrating that lean neural networks and specialized hardware can improve inference time for waveform design without sacrificing accuracy. Our lean neural solution incorporates problem-specific information into the layer structures and loss functions to decrease network size and improve accuracy. We provide model outcomes implemented on radio frequency system on a chip (RFSoC) hardware that support our simulation results. Our neural network solution decreases inference time on traditional CPU hardware by 1057× and on GPU hardware accelerators by 883× while maintaining 99% cosine similarity.

## 1. Introduction

As one of the fastest growing artificial intelligence techniques, neural networks have earned their popularity for their impressive precision at non-linear function approximation [1]. Neural networks approximate functions by training a coordinated group of tens, thousands, or millions of artificial neurons to classify input data. Neural networks have revolutionized the fields of computer vision [2], natural language processing [3], autonomous driving [4], and many others. Practitioners with sufficient quantities and quality of training data, expertise in neural network design, and computational resources can train neural networks to classify complex data with precision above and beyond human capabilities [5].

Many prior obstacles to the implementation and deployment of neural solutions, including the acquisition of large labeled datasets, compute resources for training, and a lack of software support, have been overcome in the past decade. Corporations and research institutions regularly collect and disseminate data sets for important problems which are applicable to neural networks [6,7,8]. Similarly, public and private clouds provided by the same institutions [9] and increased consumer adoption of GPUs have made neural network training more accessible. Free neural network development software packages with industry support, such as Tensorflow [10], Torch [11], and Keras [12], leverage this hardware and provide a usable interface to further facilitate neural network design and increase adoption.

Industry and academic support and increased adoption have fostered neural solutions to many problems, but not without cost. Increases in accuracy often come with increased inference time as more neurons, more complex network architectures, and thus more compute resources are required. For this reason, neural networks are often only applied to problems without classical or procedural solutions, and which can tolerate inaccuracy. Expensive hardware accelerators [13,14], creative model definitions [15], and entirely new models of computation [16] have been developed and improved to suit neural networks, which perform poorly using general purpose CPUs. Furthermore, neural networks rarely achieve 100% accuracy on even conceptually simple problems given the vague definitions of problems they solve and the variability of acceptable inputs. For example, the seemingly simple problem of hand-written digit recognition has become a common benchmark for new convolutional neural network architectures as researchers race to achieve 100% accuracy on that domains principle data set, MNIST [17].

Despite their drawbacks, neural networks are not just applicable to problems which are poorly solved by classical techniques. Some problem classes have well-defined solutions which are potentially both slower and less accurate than an optimized neural network. Online optimization algorithms, a subset of optimization algorithms that lack total knowledge of future events and instead make decisions as the input is processed, for example, can take considerable time to converge to a solution. Online optimization is used in many domains, including aerospace navigation, economic modeling, and energy systems planning [18,19,20]. One particular domain, adaptive radar waveform design, is of particular interest. Waveform design algorithms [21,22] rely on online optimization for latency-sensitive problems such as interference avoidance. As the available radio frequency (RF) spectrum saturates with interference caused by other potentially nefarious RF devices, the need for interference mitigation to keep radar applications operating at their full potential becomes more apparent. The high complexity and ensuing long convergence times for these algorithms have prompted researchers to seek alternatives. Prior work has shown that waveform design is one possible area where neural networks could greatly improve inference latency [23]. Low size, weight, and power (low SWaP) neural networks and specialized neuromorphic computing hardware for spiking neural networks have proven to be viable options; however, the goal for real-time latency without a precision drop-off has yet to be achieved.

Toward this goal, we build upon prior work and examine the trade-off between neural network accuracy and inference time when deciding model structure. We examine different intelligent neural network design methods that incorporate problem-specific information derived from the extant algorithms into the structure of the networks. This augmentation bakes problem-specific information into the network architecture and training procedure, rather than allowing a naive neural network to converge to an optimal solution over time, allowing us to shrink the size of the network without decreasing accuracy.

We present AutoWave, a neural solution for autonomous waveform design that supplies comparable precision to extant algorithms with significant latency improvements. AutoWave uses novel neural network architecture designs to maintain performance using a small network architecture tailored to the autonomous radar waveform design problem to quickly and accurately reinforce network behavior. AutoWave utilizes a custom loss function which incorporates key radar waveform metrics, such as frequency-domain phase and instantaneous frequency, as well as a unique network structure that operates on the in-phase and out-of-phase waveform signal components separately. These architecture modifications improve waveform notch depth by 3.56% over naive loss functions and model structures. AutoWave can create spectral notched waveforms 1057× faster than state of the art optimization algorithms while maintaining over 99% cosine similarity on traditional CPU hardware. AutoWave can also benefit from the highly parallelizable nature of neural networks which optimization algorithms can not, increasing performance by another 883× on GPU accelerators. We validated AutoWave in both simulation and in open-air radar experiments using a Xilinx radio frequency system on a chip (RFSoC) field-programmable gate array (FPGA), demonstrating the speed, accuracy, and versatility of AutoWave in real-world scenarios.

## 2. Background

**Figure 1:**
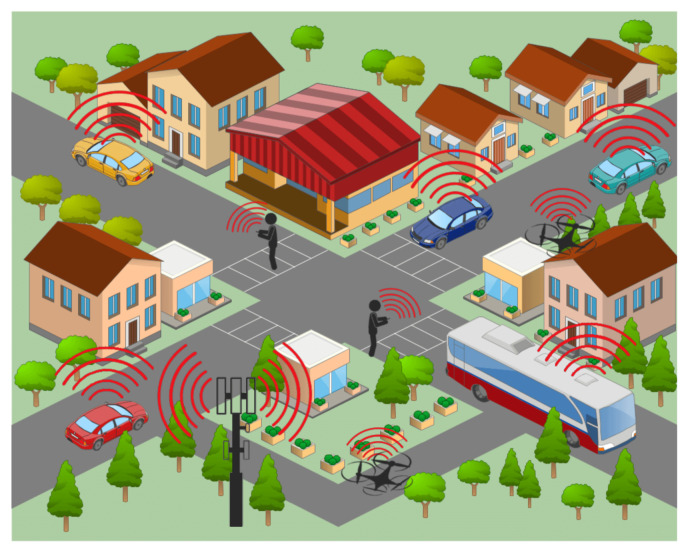
Example interference environment. Internet of Things (IoT) devices and consumer technologies, such as smartphones, transportation vehicles, drones, etc., produce radio frequency (RF) interference on an unprecedented scale.

Sources of RF interference are rapidly increasing, as shown in Figure 1, due to the growing abundance of Internet of Things (IoT) devices and consumer technologies [24]. It is therefore imperative to find ways to mitigate this interference in order for radar applications to function at maximum efficiency. We choose to focus on interference avoidance via spectral notching, a method where waveforms are modified not to transmit in the stop-band (frequencies which are saturated with interference) and instead to transmit in the pass-band where interference is less present.

**Figure 2:**
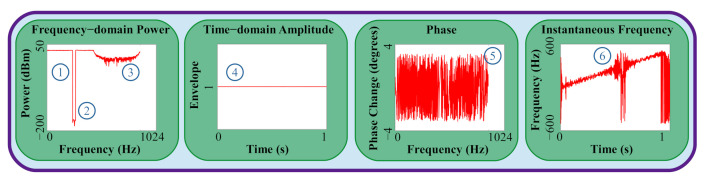
Desired waveform characteristics neural network must match: (1) Clean pass-band; (2) Deep notch in stop-band; (3) Clean roll off; (4) Constant time-domain modulus; (5) Matching phase; and (6) Notch occurring at correct time.

To be a viable solution, any output waveforms must adhere to multiple constraints as seen in Figure 2. Well-performing waveforms will have a high mean notch depth, also known as null depth, in the pass-band in order to correctly avoid interference. There should be no fluctuation in the pass-band and a well-defined roll-off should be present in the latter half of the waveform. To reduce amount of power required to transmit these waveforms and avoid unnecessary distortion of a non-linear amplifier, a constant modulus or amplitude constraint is applied to the waveforms. The relationship between the two quadrature signals, represented by the phase angle between them, should also be consistent. Lastly, the location of the notch within the time-domain must be correctly placed, which can be checked via the instantaneous frequency, to avoid the interference at the right time.

Two extant techniques are used to perform spectral notching: the Error Reduction Algorithm (ERA) [25] and the Re-Iterative Uniform Weight Optimization Algorithm (RUWO) [22]. Both of these algorithms produce spectrally notched waveforms; however, each algorithm approaches the precision-latency trade-off with different priorities.

### 2.1. Error Reduction Algorithm (ERA)

ERA, alternatively known as the Gerchberg–Saxton algorithm, is a quickly converging iterative process originally designed for reconstructing phase from intensity measurements; however this algorithm can also be used for interference avoidance after modifying the time and frequency-domain constraints. Given an object f(x) with phase components η and ψ, ERA attempts to create an estimate object $g_{k}\left(x\right)$ with matching phase components $\theta_{k}$ and $\phi_{k}$, where the subscript *k* denotes the kth iteration, by transforming between the time and frequency domains only once the constraints for that domain have been met. The 4 steps to ERA [21] are:

(1) $G_{k}\left(u\right)\;\;=\;\left|G_{k}\left(u\right)\right|\;\exp\left[i\phi_{k}\left(u\right)\right]\;=F\left[g_{k}\left(x\right)\right]$
(2) $G_{k}^{\prime }\left(u\right)\;\;=\;\left|F\left(u\right)\right|\;\exp\left[i\phi_{k}\left(u\right)\right]$
(3) $g_{k}^{\prime }\left(u\right)\;\;\;=\;\left|g_{k}^{\prime }\left(u\right)\right|\;\exp\left[i\theta_{k}\left(x\right)\right]\;=F^{-1}\left[G_{k}^{\prime }\left(u\right)\right]$
(4) $g_{k+1}\left(x\right)\;\;=\;\left|f\left(x\right)\right|\;\exp\left[i\theta_{k+1}\left(x\right)\right]\;=\;\left|f\left(x\right)\right|\;\exp\left[i\theta_{k}^{\prime }\left(x\right)\right]$

In his write-up of the ERA algorithm [21], Fienup provides an equivalence proof between ERA, in the case of the single intensity problem, and a double-length step steepest-descent search. The function being minimized is the Fourier-domain squared error

(1) $$B=\sum_{x\in \gamma }{\left[g_{k}^{{}^{\prime }}\left(x\right)\right]}^{2}$$

where γ is set of all points at which $g_{k}^{{}^{\prime }}\left(x\right)$ violates the Fourier-domain constraints, i.e., is negative or exceeds the diameter of the object. At each iteration, this method moves along the gradient from point $g_{k}$ to the point

(2) $$g_{k}^{{}^{\prime \prime }}=g_{k}\left(x\right)=h_{k}\partial_{g}B_{k}$$

with step size $h_{k}$ in the parameter space [21].

Now consider a single neuron with input vector *x*, weight matrix *w*, differentiable and non-linear activation function φ, and output value $y_{j}$ where

(3) $$y_{j}=\varphi \left(\sum_{i}w_{ij}x_{i}\right)$$

Given a target value *t*, a loss function $E=L\left(t,y_{j}\right)$ which assigns a scalar denoting the distance between the predicted and target values, and a step size η, the corresponding weight update during training is

(4) $$\Delta w_{ij}=-\eta \frac{\partial E}{\partial w_{ij}}$$

Note the similarities between Equations (2) and (4) from which we can establish a comparison between ERA and neural networks. During inference, ERA must perform a costly gradient-descent search for each waveform being passed through. However, neural networks offload these costly operations to training time, where time and power constraints are less concerning, so that inference time consists of only a handful of matrix operations. While both approaches employ convex optimization, neural networks are able to compress thousands of gradient-descent searches into a compact series of weight matrices without sacrificing accuracy, whereas ERA cannot.

### 2.2. Re-Iterative Uniform Weight Optimization Algorithm (RUWO)

RUWO is a deterministic process that performs frequency nulling using a covariance matrix

(5) $$R_{F}=Q_{F}Q_{F}^{H}+\delta I_{F}$$

and steering vector *s*. The transmitted waveforms

(6) $$S_{F}=a_{F,K}s^{T}$$

have their amplitude modulation removed with less than 50 iterations of

(7) $$a_{F,K}=\exp\left(j∠\left(R_{F}^{-1}s\right)\right)$$

Although no equivalence is provided between RUWO and convex optimization, we can define a comparison based on the computational difficulty of the RUWO algorithm. Both approaches employ the use of matrix operations; however, the size of these matrices and types of operations performed on them varies greatly between the two approaches. In RUWO, the covariance matrix has a length equal to the number of samples in the waveform, 1024 in our use case. This results in a 1024 × 1024 matrix that must be operated on for 50 iterations. In contrast, our neural networks operate on weight matrices with a maximum dimension of 1024 × 512. This is because the dimensions of the weight matrix are determined by the width of each layer in the neural network, and we attempt to keep the size of our networks small. Additionally, whereas RUWO operates for 50 iterations, our neural networks only iterate for the number of layers which, again, we limit in an attempt to reduce the SWaP of our solution. Lastly, RUWO employs the use of matrix inversion alongside multiplication to remove any amplitude modulation present in the waveforms. Comparatively, neural networks only employ matrix multiplication during inference, which is a far less intensive operation compared to matrix inversion. Overall, neural networks perform fewer and less compute-intensive operations on smaller matrices compared to RUWO.

### 2.3. Real-Time Waveform Design

Consider the example of a drone attempting to transmit radar in a crowded environment, as shown in Figure 1. An ideal solution would detect the interference in the environment, then shape a high quality waveform for real-time application on a low-power device. Figure 1 shows an ideal example of what this might look like. FPGAs would be a possible solution to the low SWaP performance constraint, while maintaining a reprogrammable structure for prototyping.

FPGAs can be implemented for real time, relatively low power application compared to CPU and GPU performance. Lean neural networks can execute on FPGA hardware with smaller form-factors and power requirements than general purpose hardware, allowing for many practical applications [26]. Specifically for RF applications, the RFSoC offers a low-SWaP, one-piece FPGA accelerated hardware for RF transmit/receive, digital signal processing (DSP), CPU control, and data transfer [27]. The versatility of this hardware allows us to compare the theoretical accuracy, speed, and power of our method to other possible algorithms such as ERA.

### 2.4. Towards Neural Waveform Design

Neural networks are powerful non-linear function approximators which construct solutions to complex problems through training. Often, neural solutions to problems with either inefficient or non-existent classical solutions rely on large training sets and standard neural network architectures [28] to quickly build sufficient classifiers. While this approach often leads to acceptable accuracy and inference time, further engineering is often required for problems with tight budgets such as radar waveform design. For domain-specific problems such as waveform design, input from subject matter experts can be used to engineer neural networks to generalize faster, prioritize important features, and decrease inference time.

In designing our network, we leveraged radar expertise to assure that our technique resulted in waveforms that prioritized waveform features that matter in communication. Specifically, we prioritized frequency-domain power, time-domain envelope, and frequency-domain phase. The frequency-domain power of our waveforms represents the amount of power transmitted at each frequency; therefore, we wish to have constant power delivered to frequencies that are not interfered and no power transmitted over frequencies that are being interfered with. Our waveforms must also maintain a constant time-domain amplitude for power efficiency due to the large power scaling caused by nonlinear radar power amplifiers. Finally, the frequency-domain phase of our waveforms, defined as the angle between the two quadrature signals, should be constant, again for power efficiency.

This resulted in two primary design choices, detailed in Section 3, that improved our networks accuracy and inference time. First, we constructed a custom loss function which prioritized the features identified by subject matter experts to more effectively train our model. Second, we identified that separating the complex representation of waveforms into two separate real outputs, each predicted by its own network, improved performance considerably. Using these two techniques identified through subject matter expertise, we were able to engineer an effective waveform design model.

## 3. Design

**Figure 3:**
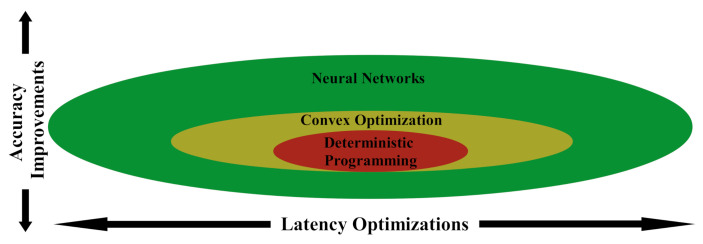
Comparison of different programming models for problem solving. The circles represent the extent of accuracy and latency improvements each programming model can achieve (i.e., a larger shape indicates more improvements exist).

To perform spectral waveform design in real time, all bloat associated with the process must be eliminated. We define bloat in this context as any functionality, inherent to the programmed solution, which prevents further accuracy or latency improvements (see Figure 3). A deterministic program for autonomous waveform design would supply the highest initial accuracy due to the specialization required to create it; however, this programming scheme offers the least accuracy and latency improvements over time due to its rigid design and difficulty programming. Convex optimization, such as the traditional algorithms ERA and RUWO, provides limited improvements over deterministic programming, but these marginal returns flatten out due to the exhaustion of hardware and software optimizations. Barring any breakthrough in convex optimization, these traditional algorithms are unable to meet real-time goals on conventional hardware, leading researchers to turn to artificial intelligence as a possible solution. Neural networks are some of the most popular artificial intelligence techniques for their portability and performance at non-linear function approximation, providing high accuracy when properly trained with sufficient data. Neural networks also, due to their inherent parallelism, boast significant continual latency improvements as new optimizations and hardware accelerators are created. However, neural networks can become saturated when the complexity of their design prevents further accuracy improvements regardless of training duration. Uninformed designs for increasing network complexity, such as increasing network depth, provide diminishing returns, as this increase in accuracy comes at the cost of higher latency times. Tailored neural network designs, such as novel modifications to the network architecture or loss function, offer better accuracy improvements without the significant increase in network latency. It is imperative that a latency-aware neural solution eliminates the need for data pre- and post-processing, as well as keeps the network depth as shallow as possible.

### 3.1. Prior Work

**Figure 4:**
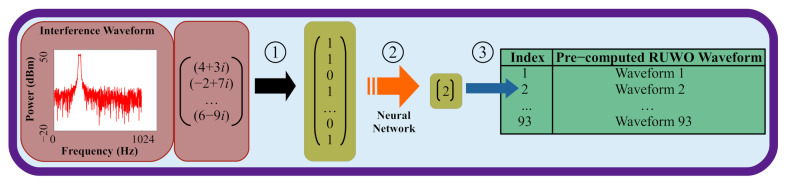
Prior neural network architectures classified input interference waveforms into pre-computed RUWO waveforms in three steps: (1) Calculate frequency-domain binary mask from complex signal, where a 0 indicates the interference was below some threshold and a 1 indicates the interference was above some threshold per Fourier bin; (2) Feed frequency-domain binary mask through neural network; and (3) Use outputted index to map to one of 93 pre-computed RUWO waveforms.

As previously mentioned, prior work [23,29,30] has shown the potential for neural network waveform design to surpass the ERA and RUWO algorithms in terms of accuracy and latency. That work showed that neural networks could be used as classifiers for indexing a lookup table of pre-computed RUWO waveforms (see Figure 4). The input for these networks was a frequency-domain binary mask which mapped each Fourier bin to a 0 or 1 based on the relation of the interference signal to some threshold. These networks were able to rapidly classify waveforms into one of 93 classes; however, the design of this solution posses numerous issues at scale.

The computation of the frequency-domain binary mask for the neural network input is a nontrivial process that requires additional Fourier transformations which increases overall network latency. This increase, although small relative to the latencies of ERA and RUWO, is considerable compared to the neural network inference time which is in the order of nanoseconds. Additionally, this input scheme requires setting an interference threshold which limits the versatility of the network.

Prior work returns a waveform class used to index into a table of pre-computed RUWO waveforms. The viability of this solution depends on the size of the RUWO table. Too small of a table would result in overlapping classifications and poor performance. Increasing the number of entries in the table would provide superior accuracy; however, that would require an increasing amount of memory on the target device. Therefore, the precision of this approach is directly tied to the size of the table which is most impacted in mobile implementations where memory is limited.

The final issue occurred when these networks were applied to direct waveform transformation where the networks themselves produced the notched waveforms rather than classify pre-computed waveforms. While the simplicity of the input resulted in impressive CPU latency, that input was not advantageous for the neural networks because it provided so little information. The resulting networks performed poorly and were unable to produce any noticeable notches compared to ERA and RUWO (see Table 1).

### 3.2. Proposed Solution

**Figure 5:**
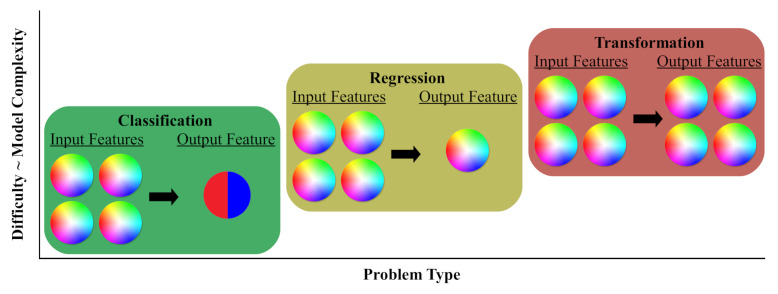
Classification problems categorize continuous input features (represented by the rainbow-colored circles) into an output class of finite size (represented by the red/blue circles). Regression allows for continuous outputs which increases the complexity of the problem. Transformation, the most difficult problem for neural networks to learn, operates similarly to regression but on a larger scale where the input and output vectors are typically the same size.

To counteract the issues with the previous work, we eliminated the need for data pre-processing and pre-computing the RUWO waveforms by having our neural networks operate directly on complex waveform data to output complex notched waveforms. This change has the adverse affect of significantly increasing the problem complexity (see Figure 5). The naive approach for counteracting this change would be to increase the model size at the expense of latency.

We found that simply increasing the depth of the networks did not drastically improve performance (see Table 2). Rather, we found a marginal 0.03% reduction in cosine similarity that came with a significant 6.5% increase in latency when growing the number of layers from one to three. The issue we were facing was a training wall that prevented in further improvements in accuracy or latency. While we had already improved upon the latency of ERA by over 200×, we wished to improve the precision and notch depth of our networks. It was clear that “off-the-shelf” approaches would not achieve this, and so we looked into more informative approaches to increase network complexity.

Our work attempts to strike a balance between model accuracy and model latency by intelligently modifying the model structure to improve accuracy without increasing latency by adding additional layers. Specifics about the model hyperparameters can be found in Section 4.2.

#### 3.2.1. Custom Loss Function

The first model structure modification we made is in the loss function of the neural network. As the driving force for determining weight updates during training, the loss function of a neural network is responsible for determining prediction error, thus teaching the neural network how to improve its predictions. This can be seen in the weight update rule (Equation (4)) where a neuron’s weights are only changed if said neuron produced an incorrect output decided by the loss function.

One common loss function, mean squared error (MSE) shown in Equation (8), performs well in a variety of situations; however, it has limitations and assumptions. MSE is generic in the sense that it does not directly pertain to any field, and so there exists the assumption that the target values are ideal because the loss function contains no helpful information. Put another way, a domain-agnostic loss function such as MSE relies solely on element-wise comparisons between the predicted output vector and the target output vector, regardless of how those vectors are constructed or represented.

(8) $$l\left(y,\hat{y}\right)\;=\frac{1}{n}\sum_{i=1}^{n}{\left(y_{i}-\hat{y_{i}}\right)}^{2}$$

In classification settings, each input image has a correct answer associated with it based on the presence or lack of phenomena. However, in transformation settings, such as spectral notching, networks return higher-dimensionality results which may not be completely correct, but sufficiently approximate correct results. This is different to regression, where the output of the neural networks is usually a small vector. These transformation scenarios consist of equally large input and output vectors which require a different approach. Neither the outputs of ERA or RUWO are necessarily perfect waveforms, rather they are different attempts to satisfy the constraints and so they are both correct to varying degrees. Training a neural network with MSE to target RUWO waveforms asserts that RUWO is ideal and that the neural network should numerically mimic the RUWO outputs. RUWO outputs are represented as coefficient vectors: a representation that solely exists for algorithm compatibility and does not inherently contain any useful quantities, i.e., the numerical difference between the coefficient vectors of the neural network output and RUWO does not properly reflect the presence of desired waveform characteristics delineated in Figure 2. Thus, without a relevant measure of waveform quality, there is no room for precise improvements. However, by implementing a custom loss function that includes these waveform characteristics, we can assure quality output waveforms after training, and potentially exceed RUWO performance.

A custom loss function that is tailored to the problem field gives neural networks a better understanding of the problem being solved. Rather than using neural networks as fast and light-weight classifiers, because neural networks are able to consolidate a large input to output table, we want to view neural networks as agents that are capable of performing the true waveform design task.

As previously discussed, the quality of spectrally notched waveforms greatly depends on multiple characteristics, and in order for the neural network to succeed in solving the interference mitigation task, its output waveforms must meet all of the characteristics. The simple approach for meeting these constraints and producing highly accurate waveforms is to drastically increase the network size. Instead, we choose to incorporate these characteristics directly into the network structure by explicitly including them in the loss function. This design choice reinforces beneficial waveform qualities to better guide the neural network toward ideal outputs while also keeping maintaining fast inference by minimizing model width and depth. Rather than performing a simple element-wise numerical comparison of the output vectors, we can now construct loss quantities that account for relations between different vector elements which ultimately produces a more efficient agent capable of focusing on not just the numerical output, but on the necessary characteristics of a successful waveform.

Our custom loss function between the actual waveform *y* and the predicted waveform $\hat{y}$, where $y,\hat{y}\in R^{2n}$ are coefficient vector representations of the respective frequency-domain waveforms, is shown in Equation (9). Note that $z,\hat{z}\in C^{n}$ are the re-combined complex representations of the respective frequency-domain waveforms which are used to calculate the frequency-domain power, time-domain envelope, and frequency-domain phase.

(9) $$\begin{array}{cc} l\left(y,\hat{y}\right) & =\frac{1}{2n}\sum_{i=1}^{2n}{\left(y_{i}-\hat{y_{i}}\right)}^{2}\\ & +\frac{1}{n}\sum_{i=1}^{n}{\left(20*\log_{10}\left(|z_{i}|\right)\;-\;20*\log_{10}\left(|\hat{z_{i}}|\right)\right)}^{2}\\ & +\frac{1}{n}\sum_{i=1}^{n}{\left(IFFT\left(|z_{i}|\right)\;-\;IFFT\left(|\hat{z_{i}}|\right)\right)}^{2}\\ & +\frac{1}{n}\sum_{i=1}^{n}{\left(∠|z_{i}\left|-∠\right|\hat{z_{i}}|\right)}^{2} \end{array}$$

As traditional neural network loss functions operate only on real values, the default MSE loss function can only operate on the coefficient vector which loses important information. Our custom loss function metrics “re-combine” the real and complex signals when computing the loss which saves that previously lost information. Additionally, our custom loss function allows for metrics which take into account relationships between different elements rather than just element-wise comparisons. With a problem as complex as autonomous radar waveform design where each point of a waveform must not only be numerically correct, but also correct in relation to the other points of waveform, it is important to capture all aspects of the waveform in the neural network learning process.

#### 3.2.2. Separate I and Q Models

**Figure 6:**
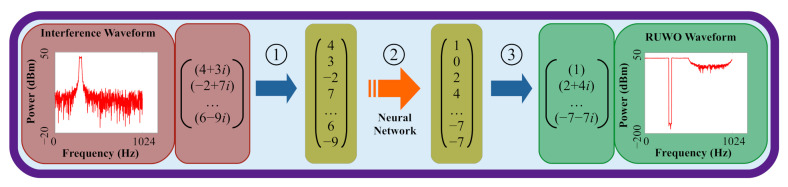
Our neural network architecture transforms input interference waveforms directly into transmittable notched waveforms in three steps: (1) Convert complex signal into coefficient vector for neural network framework compatibility; (2) Feed coefficient vector through neural network; and (3) Convert new coefficient vector into complex signal.

Our second design improvement focuses on the specific structure of the radar interference mitigation problem. The nature of this problem revolves around two real-valued signals in quadrature where their representation as one complex-valued signal is mathematical shorthand for human comprehension. One of the issues that stems from this representation is the necessary splitting of complex data into coefficient vectors for neural network processing because these models operate on real values as shown in step (2) in Figure 6.

This representation conflicts with the behavior of the signals as touched upon earlier. Consider the scenario of image processing where human eyes are naturally able to view two-dimensional images. Naive neural networks, on the other hand, are unable to process the array of pixel values and are forced to flatten the pixels into a vector representation. This representation is inappropriate because relationships between neighboring pixels are lost. The emerging solution for this issue was the addition of convolutions where many small sub-matrices of the image are added as features to the network to leverage data locality. This method still produces vectors; however, the spatial pixel relationships are encoded into the vectors which give convolutional neural networks an advantage for image processing.

**Figure 7:**
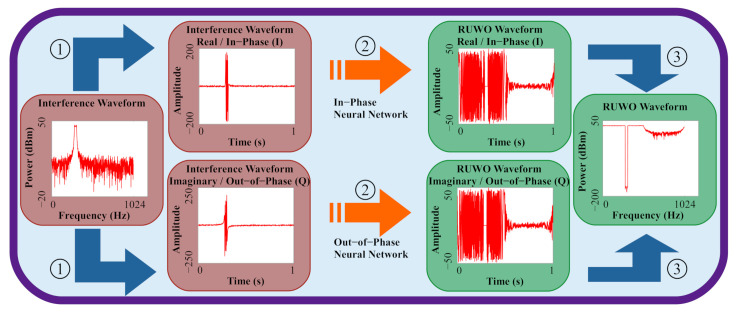
Our split neural network architecture transforms input interference waveforms directly into transmittable notched waveforms, similar to the traditional neural network architecture, but now does so in three parallel stages: (1) Split complex signal into the two corresponding real-valued signals I and Q; (2) Feed real signals through two separate neural networks; and (3) Combine new real-valued signals into complex signal.

Our novel idea is to separate the complex signal into the two corresponding real signals that are fed into two different neural networks (Figure 7). Separating the two quadrature signals broke the problem into designing a single real-valued waveform where a numerical element-wise comparison now corresponds with the quality of an object that exists beyond its representation, i.e., the real-valued signal has directly applicable qualities, whereas the coefficient vector containing both quadrature signals does not. Each of these neural networks then outputs the corresponding real signals which are then combined to form the final complex output signal. Note that the waveforms are split and later recombined in the frequency domain for information compression. By keeping the real signals intact, the information pertaining to the waveform is also preserved throughout the network because the network is learning the problem in such a way that does not depend on the data’s representation.

## 4. Materials and Methods

In this section, we provide details for the data generation, algorithm setup, and neural network implementation toward replication and advancement of our work. We include the specific hardware used for both simulation and our open-air trials as well as any software libraries, and their respective parameters, used for machine learning.

### 4.1. Data Generation

Our data generation scripts, which include ERA and RUWO implementations, were coded in MATLAB version 2021a. A sampling frequency of 1024 Hz with a transmit bandwidth of 512 Hz was used to generate the linear frequency modulated (LFM) interference signal matrix that was used as input for the ERA and RUWO algorithms as well as the neural networks. Our dataset consisted of 262,144 input LFM interference and corresponding output RUWO waveforms. Gaussian noise with an amplitude of 0.1 and variance 1 was used to provide variety in the training dataset. Data generation occurred on a standard memory node on the Mustang HPE SGI 8600 system, a U.S. Air Force Research Laboratory (AFRL) DoD Supercomputing Resource Center (DSRC) machine, powered by dual Intel Skylake Xeon 8168 CPUs and 192 GB of RAM.

### 4.2. Neural Network

Our neural network models were implemented in Python 3.6.8 using the Keras 2.3.1 library and the Tensorflow 2.2.0 machine learning back-end library. The Hyperas 0.4.1 library, a Hyperopt wrapper for Keras models, was selected for performing the hyperparameter optimization. Training occurred on a GPU node on the Mustang HPE SGI 8600 system, a U.S. Air Force Research Laboratory (AFRL) DoD Supercomputing Resource Center (DSRC) machine, powered by dual Intel Skylake Xeon 8168 CPUs, 384 GB of RAM, and a NVIDIA Tesla P100 GPU for neural network acceleration. Trainable hyperparameters included layer depth, layer width, dropout rate, activation function, and the loss function. We used the tree-structured Parzen estimator (TPE) in Hyperas for hyperparameter optimization, and our selected hyperparameters for all models can be found in Table 3. We used K-Fold cross-validation using 10 folds and cosine similarity to evaluate training progress where each training trial lasted for 100 epochs. Standard error across the 10 folds are provided along with the mean value for replication.

#### Split IQ Model

The split IQ models were implemented using the Keras functional API which allows for more custom network architectures. Contained within these models are two neural networks that operate on the in-phase (real) and out-of-phase (imaginary) components of the waveform separately. These sub networks are run simultaneously, and each sub network uses the same hyperparameters discussed above.

### 4.3. RFSoC

**Figure 8:**
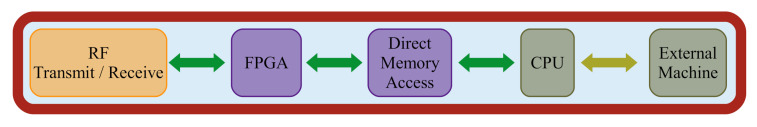
Radio frequency system on a chip (RFSoC) structure: field-programmable gate array (FPGA) sends and receives data from the RFSoC and direct memory access (DMA). The accompanying CPU resources can read and write to the DMA and communicate with another machine via wired connection.

**Figure 9:**
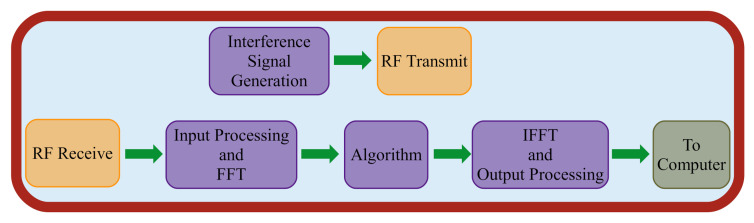
Data flowchart of FPGA.

All algorithms were implemented using Simulink with MATLAB R2020a and the hardware description language (HDL) generation toolbox. We generated the HDL code using Vivado v2019.1 which includes optimized HDL-code blocks for functions such as FFT/IFFT and tanh which benefited our algorithms. All tests were run on the Xilinx ZCU111 RFSoC with a FPGA clock rate of 128 MHz (the architecture of which can be seen in Figure 8 and Figure 9). Note that floating point values are not supported, so the weights and biases are quantized to signed 18-bit fixed point values before being sent to the RFSoC.

We used the same hardware and non-algorithm sections of the HDL for all comparisons. The input signal for our ERA, RUWO, and NN model implementations was received as a 1024 sample, 18 bit, 16 fractional signed complex fixed point input with a single interference band. This signal was generated using a LFM chirp that sweeps through a range of frequencies and appears as a band of interference in the frequency spectrum. After passing through the appropriate HDL-code blocks of our algorithms, the output is an interference mitigated signal with the same sample size and datatype that is ready for transmission back to the computer for analysis.

## 5. Results

In this paper, we defined the trade-off between speed and precision and the necessity to choose intelligent structures for solving the autonomous waveform design problem. We now show that our neural network solutions to this problem produce comparable precision to the leading convex optimization algorithms with drastic latency improvements. We also show that incorporating problem-specific information into the neural network structure to create agents capable of performing radar waveform design produces better yielding networks as opposed to “off-the-shelf” neural networks which rely on approximations stemming from uninformed memorization.

We first explore the correctness of our neural network solution in the context of cosine similarity (see Table 4). Our neural networks operate within 2.2% cosine similarity compared to the RUWO algorithm and within 1.2% compared to the ERA algorithm. As the defining metric for spectral waveform quality, the null depth results show that our neural networks are capable of performing waveform design with satisfactory precision. Furthermore, we show that our neural network tailoring towards waveform design produced better performing networks that were able to beat “off-the-shelf” networks in null depth by 4.24%. From this, we can assert that neural networks are a suitable replacement to these algorithms.

**Figure 10:**
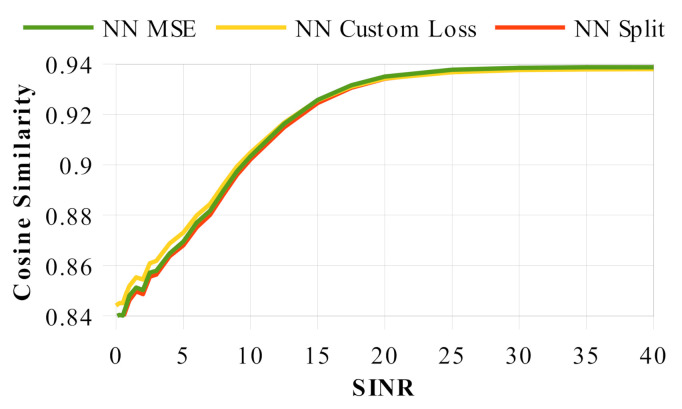
Neural network cosine similarity across a range of varying environments as defined by the signal to interference plus noise ratio (SINR).

While demonstrating our neural network solutions ability to correctly perform the autonomous waveform design problem, we also provide evidence of the robustness of our solution. We explored the model performance as we modified the input waveforms (which can be found in Figure 10) and found that our models were continually able to succeed in their task regardless of the input waveform quality, thus showing the models ability to generalize in a broad spectrum of RF environments.

**Figure 11:**
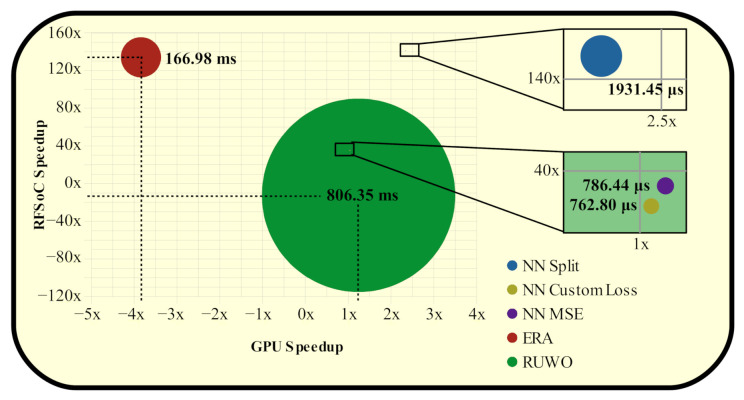
Convex optimization and neural networks CPU latencies (shown in bold) along with GPU and RFSoC accelerator speedups. Latencies are represented as the size of the circles, with the circles centered at their respective GPU and RFSoC accelerator speedup values.

We also show that these neural networks achieve their precision with significant latency improvements, three orders of magnitude, compared to convex optimization on traditional CPU hardware (see Figure 11). Such drastic reduction in latency allows for radar waveform design to broaden its application-space, especially for mobile usage. Additionally, we provide latency comparisons on multiple hardware accelerators, such as the NVIDIA Tesla P100 GPU and the Xilinx XZU111 RFSoC which utilizes an FPGA, which also demonstrate significant speedup over convex optimization (see Table 5). The portability of neural networks onto a variety of different hardware platforms, with little overhead, allows researchers to broaden waveform design applications to spaces that would otherwise be impossible to implement due to time and power constraints, such as mobile development.

### 5.1. Raspberry Pi

We also provide latency results on the Raspberry Pi platform (see Table 6). We use the low-cost Raspberry Pi 3 Model B, which features a quad core 1.2 GHz Broadcom BCM2837 64-bit CPU and 1 GB of LPDDR2 RAM but has a small credit-card sized profile. We show that our neural network approach delivers faster results not only on high-end accelerators, but also on low-end consumer-grade embedded hardware.

### 5.2. RFSoC Open Air Trials

Aside from providing an additional hardware accelerator comparison, we also use the RFSoC for open air trials to validate our simulation results. As previously mentioned, the training and testing data for the neural network development was generated using simulations. To demonstrate the robustness of our solution, we used the RFSoC to physically transmit the interference waveforms in order to better test out neural network solution in a more practical environment.

In the open air trials, we found that our neural network approach created quality waveforms that conformed to the desired characteristics. Specifically, our neural network produced waveforms with a notch depth within 17% compared to the RUWO algorithm (see Table 7). These results show that our neural network approach performs satisfactorily in both simulation and real-world application.

**Figure 12:**
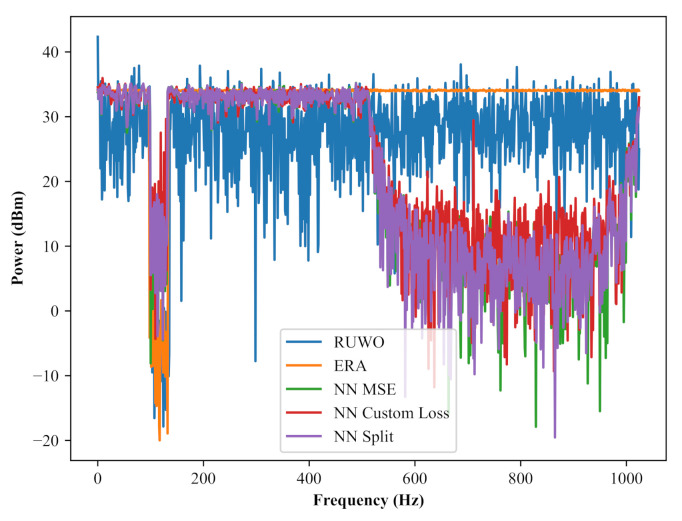
Sample waveforms generated on the RFSoC hardware.

While performing the open air trials, we discovered limitations with the RUWO algorithm implemented on the RFSoC hardware. The FPGA hardware of the Xilinx XZU111 RFSoC requires all variables to be stored as fixed point values as opposed to traditional floating point storage found on CPU and GPU hardware. This restriction reduces the granularity of variable representation which, when coupled with the exceptionally high precision the RUWO algorithm expects, produces much lower quality waveforms as seen in Figure 12. Note that the neural network and ERA implementations did not reduce so drastically in quality from this hardware limitation and were able to produce waveforms consistent with their corresponding CPU/GPU implementations. From this, we can determine that neural networks have better resiliency to different hardware platforms.

## 6. Discussion

We have demonstrated the effectiveness of neural networks applied to the autonomous radar waveform design problem, pioneering the usage of AI in radar problems. We achieved speedups of over 1000× with less than 2.2% drop in cosine similarity compared to traditional algorithms, showing the viability of neural networks applied to other radar and waveform design fields where current solutions are also hindered by poor performance times. We have shown the effectiveness of our neural network solution on different specialized hardware, thus allowing easier portability of our solution to lower cost accelerators compared to the difficulty and cost of implementing traditional algorithms on application-specific integrated circuits (ASICs). We have also shown, through our Raspberry Pi experiments, that our neural network solution can take on a wider array of applications where traditional algorithms would be inappropriate due to time or power constraints. For example, cars and low-power devices can now benefit from these radar applications running on native hardware and low-cost commercial “off-the-shelf” hardware [31]. The speedups achieved and low SWaP nature of neural networks opens the door for different radar applications where existing algorithms would be an obstacle.

## 7. Conclusions

Recent advances in the neural network domain, such as increased availability of high-quality data sets and improvements in development libraries, have drawn researchers towards using neural networks as viable solutions in different domains. Computer vision, natural language processing, and autonomous systems have all benefited from the parallelizable non-linear function approximation neural networks excel in performing. Traditionally, neural networks were applied to a small subset of problems because they were considered too slow and imprecise. However, prior work shows that neural networks could be viable replacements for algorithmic solutions. That work focused on the autonomous waveform design problem, a conflict against RF interference produced by consumer and commercial technologies in order to keep radar applications operating at peak efficiency. Researchers found that neural networks performed satisfactorily with significant latency improvements over the established convex optimization algorithms, opening the door for this work which aimed to show the viability of neural networks as stand-alone replacements for slow-converging convex optimization algorithms.

Building on that previous work, we demonstrated that low SWaP neural networks can better utilize specialized parallel hardware and improve latencies for autonomous waveform design without compromising accuracy. Taking an additional step, we found that augmenting off-the-shelf neural network architectures with structures stemming from the radar design space yielded higher network accuracy with a smaller network size.

Future work should apply specialized network design to other radar and optimization domains, including those which do not have existing solutions. Additional waveforms, characteristics, and communication models should be analyzed along with their power and compute requirements. Furthermore, performance on real-world radar communication tasks should be evaluated more fully. Custom evaluation metrics should be devised to provide better insight into neural network performance and complement the custom loss function used during training. This, in addition to researching algorithm decomposition for greater network architecture customization, will broaden the application of specialized network design and create stronger tailored solutions.

We showed that our neural network approach could perform autonomous waveform design within 2.2% cosine similarity of the leading convex optimization algorithms with drastic latency improvements of over 1000×. With sufficient training and thorough design, neural networks can be viable replacements for convex optimization algorithms.

## Figures and Tables

**Table 1:** Comparison of convex optimization algorithms RUWO and ERA to initial AutoWave neural networks (NN) modified to output notched waveforms rather than classify pre-computed waveforms.

Algorithm	CPU Latency (μs)	Cosine Similarity	Null Depth (dBm)
RUWO	782,800.0 ± 0.0	1.0 ± 0.0	202.22 ± 0.0
ERA	163,420.0 ± 0.0	0.9982 ± 0.0	31.89 ± 0.0
NN Binary Mask MSE	637.4 ± 4.57	0.7755 ± 1.81 $\times \;10^{-4}$	0.0 ± 0.0
NN Binary Mask Custom Loss	632.9 ± 4.83	0.7649 ± 2.91 $\times \;10^{-3}$	0.0 ± 0.0

**Table 2:** Drawback of naively increasing number of layers to maintain cosine similarity in higher complexity problem.

Algorithm	GPU Latency (μs)	CPU Latency (μs)	Cosine Similarity	Null Depth (dBm)
NN MSE 1 Layer	747.71 ± 5.23	786.44 ± 5.01	0.9901 ± 7.69 $\times \;10^{-5}$	28.54 ± 0.16
NN MSE 2 Layers	749.40 ± 5.61	797.92 ± 5.99	0.9900 ± 7.89 $\times \;10^{-5}$	29.17 ± 0.22
NN MSE 3 Layers	797.72 ± 10.03	855.34 ± 6.38	0.9898 ± 9.87 $\times \;10^{-5}$	26.57 ± 0.23

**Table 3:** Hyperparameter values used for our neural networks.

Hyperparameter	Value
Network Depth (# layers)	1
Network Width (# neurons)	256
Dropout Rate	0.2
Activation Function	tanh

**Table 4:** Comparison of correctness between convex optimization and our neural network approaches.

Algorithm	Cosine Similarity	Null Depth (dBm)
RUWO	1.0 ± 0.0	202.23 ± 0.0
ERA	0.9982 ± 0.0	31.89 ± 0.0
NN MSE	0.9901 ± 7.69 $\times \;10^{-5}$	28.54 ± 0.16
NN Custom Loss	0.9789 ± 9.53 $\times \;10^{-5}$	22.32 ± 0.13
NN Split	0.9900 ± 1.08 $\times \;10^{-4}$	29.75 ± 0.12

**Table 5:** Comparison of accelerator utilization amongst the different approaches.

Algorithm	CPU Latency (μs)	GPU Latency (μs)	RFSoC Latency (μs)
RUWO	806,347.0 ± 11,860.82	649,581.0 ± 33,168.78	10,060,000.0 ± 999.0
ERA	166,982.0 ± 3465.06	641,441.0 ± 20,921.13	1246.0 ± 8.8
NN MSE	786.4 ± 5.01	747.71 ± 5.23	21.7 ± 0.0
NN Custom Loss	762.8 ± 5.04	735.68 ± 7.99	21.7 ± 0.0
NN Split	1931.5 ± 9.75	823.63 ± 6.76	13.7 ± 0.0

**Table 6:** Latency measurements using the low-power Raspberry Pi platform.

Algorithm	Raspberry Pi Latency (s)
RUWO	459.061 ± 4.741
ERA	1.953 ± 0.039
NN MSE	0.230 ± 0.003
NN Custom Loss	0.230 ± 0.003
NN Split	0.257 ± 0.003

**Table 7:** Notch depth comparison on FPGA hardware.

Algorithm	Null Depth (dBm)
RUWO	33.62 ± 0.041
ERA	37.13 ± 0.057
NN MSE	28.17 ± 0.213
NN Custom Loss	21.22 ± 0.138
NN Split	28.93 ± 0.285

## Data Availability

Not applicable.
